# Cephalometric traits of class II division 2 mal-occlusion among Bengalis in India

**DOI:** 10.6026/973206300210549

**Published:** 2025-03-31

**Authors:** Kasturi Mukherjee, Shyamal Bar, Prakash Banerjee, Poulomi Roy, Kasturi Mukherjee

**Affiliations:** 1 Department of Orthodontics and Dentofacial Orthopedics' at Burdwan Dental College and Hospital, India

**Keywords:** Class II div II malocclusion, lateral cephalogram, digital and manual tracing, skeletal and dental parameters, comparison with class i malocclusion

## Abstract

Numerous cephalometric studies have been conducted to assess the features of class II division 2 malocclusions since the release of Angle's description of malocclusion types; nonetheless, disagreements persist. Therefore, it is of interest to examine the cephalometric features of Class II/2 malocclusions in patients aged 8 to 24 at Burdwan Dental College and Hospital, given that a significant proportion of patients, including adults seeking orthodontic treatment, have this malocclusion. Each lateral cephalogram was traced both digitally and manually using the Nemo Ceph Software System. Thirty-six dental and skeletal parameters were measured; thirty class II Division 2 participants (15 girls and 15 boys) and thirty subjects with normal occlusion were chosen and clinically assessed.

## Background:

Understanding the skeletal and dental features of a certain malocclusion is crucial when treating orthodontic disorders since it might affect our treatment strategy. Accurate diagnosis is the first step in any successful orthodontic therapy. Numerous combinations of skeletal and dentoalveolar components can lead to class II malocclusion, a prevalent form of malocclusion [1]. Class II/2, which accounts for 1.5% to 7% of all malocclusions in the white western population, is comparatively uncommon among them [2]. Several studies have considered the components of class II division2 malocclusion, but there was not an agreement on skeletal or dental imbalances. According to some researchers, with the exception of retroclined upper incisors, class II/2 has not a particular skeletal pattern when compared to class II/1 5-9 but others showed that this malocclusion is a distinct dento-skeletal deformity [3]. Some studies have found no maxilla-mandibular dentoalveolar discrepancy [4]. However, Pancherz *et al.* (1997) [5] stated that mandibular retrusion was a common characteristic not only of class II division 1 subjects but also of division 2 subjects. This controversy might be the result of sample size, sample selection criteria, age range, the cephalometric points identified and the types of statistical tests used. Class II/2 malocclusion is usually associated with an increased posterior facial height 13, a reduced mandibular plane angle a reduced anterior facial height, a more horizontal growth vector, prominent chin, retroclined incisors and class II molar relationship [6]. Therefore, it is of interest to examine the cephalometric features of class II/2 malocclusions in patients aged 8 to 24 at Burdwan Dental College and Hospital, given that a significant proportion of patients, including adults seeking orthodontic treatment, have this malocclusion.

## Methods and Materials:

Clinical examinations were performed on the patients at Burdwan Dental Collage and Hospital. Of them, 30 patients aged 8-20 years with class II/2 malocclusion and 30 patients with normal occlusion were chosen as a control group. The following were the requirements for class II/2 inclusion:

[1] No prior orthodontic treatment history

[2] Deep bite with retroclination of the maxillary front teeth (at least two central incisors). After two specialists agreed, all cases were accepted.

There were 15 females and 15 boys in class I, ages 10.4+1.3, and class II/2, respectively, with ages ranging from 10.24+1.22. Twenty dental and skeletal characteristics were measured, each lateral cephalogram was traced both manually and digitally using the Nemo Ceph Software System, and all individuals were chosen and clinically evaluated. On each patient's lateral cephalogram, cephalometric landmarks were noted. A pair of orthodontists determined each milestone. The markers of cephalometry were later digitalized.

## The following skeletal parameters were used:

## Sagittal variables:

Sella-nasion to A-point angle, Sella-Nasion-B point, A point-Nasion-B point angle, facial angle, angle of convexity.

## Vertical variables:

Y-axis angle, mandibular plane angle, palatal-mandibular plane angle, N-ANS, ANS-Me, gonial angle

## Mandibular measurement:

Ramus height (Ar-Go), body length (Go-Gn)

## Dental parameters were used:

Lower one to mandibular plane, lower one to occlusal plane, lower one to NB (mm), lower one to NB (angle), upper one to NA (mm), upper one to NA (angle), and upper one to A-pog. Two months later, they were tracked in order to investigate the measurement mistake. Two measures did not vary statistically significantly, according to the paired T-test. Dahlberg's (1940) formula was used to determine that all measurement error coefficients were within acceptable bounds and larger than 0.90.

## Statistical analysis:

Graph Pad Prism version 5 and SPSS (version 27.0; SPSS Inc., Chicago, IL, USA) were used to evaluate the data after it was entered into a Microsoft Excel spreadsheet. The data was summarized using count and percentages for categorical variables and mean and standard deviation for numerical variables. There were two sample t-tests for a mean difference that used either unpaired or independent samples. Blocking was done with paired t-tests, which were more powerful than unpaired tests. Any statistical hypothesis test in which, in the case of a true null hypothesis, the sampling distribution of the test statistic is a chi-squared distribution is known as a chi-squared test (χ^2^ test). The term 'chi-squared test' is frequently used as shorthand for Pearson's chi-squared test without any more explanation. Fischer's exact test or the Chi-square test, if applicable, was used to compare unpaired proportions. The following list contains explicit expressions that may be used to perform different t-tests. The formula for a test statistic that closely resembles or perfectly matches a t-distribution under the null hypothesis is provided in each instance. In every instance, the proper degrees of freedom are also provided. It is possible to do a one-tailed or two-tailed test using each of these statistics. After a t value has been established, a table of values from the Student's t distribution may be used to determine a p-value. In the event that the computed p-value falls below the statistical significance threshold (often the 0.10, 0.05, or 0.01 level), the alternative hypothesis is accepted and the null hypothesis is rejected. P-values < 0.05 were regarded as statistically noteworthy.

## Results:

In Cl II Div II group, the mean sella-nasion to A-point angle (d) (mean± sd.) of patients was 77.528±0.602. In Normal Occlusion group, the mean sella-nasion to A-point angle (d) (mean± sd.) of patients was 80.32±0.915. Distribution of mean sella-nasion to a-point angle (d) with both procedure was statistically significant (p<0.0001) (Table 1). In Cl II Div II group, the mean Sella-Nasion-B point (d) (mean± sd.) of patients was 71.59±0.912. In Normal Occlusion group, the mean sella-Nasion-B point (d) (mean± s.d.) of patients was 78.12±0.927. Distribution of mean Sella-Nasion-B point (d) with both procedure was statistically significant (p<0.0001) (Table 2). In Cl II Div II group, the mean 1 To MPA (d) (mean± sd.) of patients was 5.197±0.193. In Normal Occlusion group, the mean 1 To MPA (d) (mean± s.d.) of patients was 6.763±0.524. Distribution of mean 1 To MPA (d) with both procedure was statistically significant (p<0.0001 (Table 3). In Cl II Div II group, the mean 1 To OPA (d) (mean± s.d.) of patients was 17.217±0.716. In Normal Occlusion group, the mean 1 To OPA (d) (mean± s.d.) of patients was 24.507±0.758. Distribution of mean 1 To OPA (d) with both procedure was statistically significant (p<0.0001) (Table 4). In Cl II Div II group, the mean 1 TO NB (<) (mean± s.d.) of patients was 6.267±0.357. In Normal Occlusion group, the mean 1 TO NB (<) (mean± sd.) of patients was 25.983±1.521. Distribution of mean 1 TO NB (<) with both procedure was statistically significant (p<0.0001) (Table 5). In Cl II Div II group, the mean 1 TO NB (mm) (mean± s.d.) of patients was 1.676±0.194. In Normal Occlusion group, the mean 1 TO NB (mm) (mean± sd.) of patients was 5.157±0.676. Distribution of mean 1 TO NB (mm) with both procedure was statistically significant (p<0.0001) (Table 6).

## Discussion:

The finding of this study revealed that Class II division 2 malocclusion is not a single clinical entity. To facilitate reading, cephalometric measures will be discussed in topics: The sella-nasion to A-point angle in the class II division 2 sample was lower than that of normal occlusion (p<0.0001). Numerous additional investigations have indicated that class II/2 malocclusion patients have a slightly protruded [7] position, making this conclusion extremely contentious. However, our results were in line with Pancherz's, who observed that the sella-nasion to A-point angle was lower in his class II/2 samples when he compared them to the reference data from Michigan and London. Comparing the mandibles of individuals with Class II/2 malocclusions to those with Class I occlusions, the literature often describes the former as retrognathic [8]. These conclusions are supported by the current study's results. SNPg and Sella-Nasion-B point angle were both much lower in class II division 2 samples. In Class II/2 malocclusion, the mandibular sagittal position has an intermediate value, according to other research [9], although Blair [7] reported a somewhat prognathic mandible. In Class II/2 malocclusion, Renfroe [10] observed a somewhat longer mandible, but Kerr *et al.* [9] and Kerr and Adams [11] did not observe any distinction in the mandibular morphology between Class II/1 and Class II/2 malocclusions. A pattern like that of Sella-Nasion-B point and SNPg was discovered when sagittal mandibular position was assessed using Pg-Nperp. In class II division 2 samples, the A point-Nasion-B point angle angle was larger than that of normal occlusion (p<0.0001). Similar to our results, Hitchcock and Pancherz observed a statistically significant increase in the A point-Nasion-B point angle angle in Class II/2 malocclusions [12] with regard to the intermaxillary connection. Class II/2 samples exhibited a significantly higher angle of convexity than class I occlusion (p<0.01), but the variations in facial angles between the two were not statistically significant. The vertical characteristics of Class II/2 malocclusion include a flat mandibular plane, an acute gonial angle, an enlarged posterior facial height, a reduced anterior facial height, and a more horizontal growth vector as indicated by the Downs Y-axis and Ricketts facial axis. The preceding list describes a definite hypodivergent facial pattern in the Class II/2 malocclusion group.

The study observed various cephalometric differences between Class II/2 malocclusion and normal occlusion. The inclination angle showed no significant change (P=0.055), while the Y-axis was notably smaller in Class II/2 samples (P<0.0001), indicating a significant difference from Class I occlusion. Class II/2 patients also had a narrower mandibular plane angle (P<0.0001) and a more acute palatal-mandibular plane angle, consistent with prior research. The N-ANS measurement showed no significant difference (P=0.66), but the ANS-Me distance, reflecting lower anterior face height, was significantly reduced in Class II/2 (P<0.001), aligning with Karlsen's findings. The gonial angle was smaller in Class II/2 patients (P<0.0001), and mandibular measurements such as ramus height and body length were larger in these samples (P<0.0001), contradicting some earlier studies that described the mandible in Class II/2 patients as smaller. In terms of dentoalveolar features, the study confirmed characteristics like deep overbite, obtuse interincisal angle, and retroclination of the upper central incisors, consistent with previous research on Class II/2 malocclusion. The lower incisors were found to be retroclined to the Frankfort Horizontal, while maintaining normal inclination to the mandibular plane and NB, providing insight into seemingly contradictory findings in the literature.

## Conclusion:

There are several distinctive cephalometric characteristics in class II/2 malocclusion compared to class I normal occlusion. The maxilla is positioned further back, or retruded, while the mandible, despite its greater overall length, is also retruded in the sagittal plane. This results in a sharp gonial angle, which is a notable feature. The overall facial structure tends to follow a hypodivergent growth pattern, primarily due to a horizontal mandibular growth vector. This causes a flat mandibular plane and excessive skeletal counterclockwise rotation of the mandible. Additionally, both the upper and lower incisors are retroclined, contributing to a deep overbite that is primarily skeletal in origin rather than being driven by dentoalveolar factors. This skeletal pattern in Class II/2 malocclusion sets it apart from class I occlusion, where the bite and facial structure are more balanced and typically characterized by more upright incisor positioning and a different growth pattern.

## Figures and Tables

**Table 1:** Distribution of mean sella-nasion to A-point angle (d): Group

		**Number**	**Mean**	**SD**	**Minimum**	**Maximum**	**Median**	**P-value**
SELLA-NASION TO A-POINT ANGLE (d)	Cl II Div II	30	77.528	0.602	76	78.83	77.75	<0.0001
	Normal Occlusion	30	80.32	0.915	78	82	80	

**Table 2:** Distribution of mean Sella-Nasion-B point (d): Group

		**Number**	**Mean**	**SD**	**Minimum**	**Maximum**	**Median**	**P-value**
SNB (d)	Cl II Div II	30	71.59	0.912	70	73	72	<0.0001
	Normal Occlusion	30	78.12	0.927	76	80	78	

**Table 3:** Distribution of mean 1 To MPA (d): Group

		**Number**	**Mean**	**SD**	**Minimum**	**Maximum**	**Median**	**P-value**
1 To MPA (d)	Cl II Div II	30	5.197	0.193	5	5.5	5.2	<0.0001
	Normal Occlusion	30	6.763	0.524	5	7.6	7	

**Table 4:** Distribution of mean 1 To OPA (d): Group

		**Number**	**Mean**	**SD**	**Minimum**	**Maximum**	**Median**	**P-value**
1 To OPA (d)	Cl II Div II	30	17.217	0.716	15.8	18.3	17.15	<0.0001
	Normal Occlusion	30	24.507	0.758	23	25.4	25	

**Table 5:** Distribution of mean 1 TO NB (<): Group

		**Number**	**Mean**	**SD**	**Minimum**	**Maximum**	**Median**	**P-value**
1 TO NB (<)	Cl II Div II	30	6.267	0.357	5.1	7	6.2	<0.0001
	Normal Occlusion	30	25.983	1.521	22	27.8	26	

**Table 6:** Distribution of mean 1 TO NB (mm): Group

		**Number**	**Mean**	**SD**	**Minimum**	**Maximum**	**Median**	**P-value**
1 TO NB (mm)	Cl II Div II	30	1.676	0.194	1.2	2.1	1.675	<0.0001
	Normal Occlusion	30	5.157	0.676	4	6	5	
